# High CD3 and ICOS and low TIM-3 expression predict favourable survival in resected oesophageal squamous cell carcinoma

**DOI:** 10.1038/s41598-019-56828-7

**Published:** 2019-12-27

**Authors:** Min Hee Hong, Su-Jin Shin, Sung Kwan Shin, Dae Joon Kim, Jae Ill Zo, Young Mog Shim, Seung Eun Lee, Byoung Chul Cho, Seong Yong Park, Yoon-La Choi, Hye Ryun Kim

**Affiliations:** 1 Division of Medical Oncology, Department of Internal Medicine, Yonsei Cancer Center, Severance Hospital, Yonsei University College of Medicine, Seoul, Korea; 2 Department of Pathology, Gangnam Severance Hospital, Yonsei University College of Medicine, Seoul, Korea; 3 Division of Gastroenterology, Department of Internal Medicine, Yonsei University College of Medicine, Seoul, Korea; 4 Department of Thoracic and Cardiovascular Surgery, Yonsei University College of Medicine, Seoul, Korea; 5 Department of Thoracic and Cardiovascular Surgery, Samsung Medical Center, Sungkyunkwan University School of Medicine, Seoul, Korea; 6 Department of Pathology, Konkuk University Medical Center, Konkuk University School of Medicine, Seoul, Republic of Korea; 7 Department of Pathology and Translational Genomics, Samsung Medical Center, Sungkyunkwan University School of Medicine, Seoul, Korea

**Keywords:** Oesophageal cancer, Tumour biomarkers

## Abstract

With the increasing oncological potential of immunotherapy, several immune checkpoint modulators are being investigated. The value of immune markers, including programmed cell death ligand-1, programmed cell death-1 (PD-1), inducible co-stimulator (ICOS), lymphocyte activation gene-3, T-cell immunoglobulin, and mucin-dominant containing-3 (TIM-3), is not well known. Using tissue microarrays of 396 patients who underwent surgery for oesophageal squamous cell carcinoma (ESCC), infiltrated T-cell subsets (CD3, CD8, and Foxp3) and checkpoint protein expression were scored. With a median follow-up of 24.8 months, CD3^+^ TIL subsets (50.0%) had longer median recurrence-free survival (RFS, 55.0 vs 21.4 months) and overall survival (OS, 77.7 vs 35.8 months). Patients with high ICOS expression (46.5%) had longer median RFS (53.9 vs 25.3 months) and OS (88.8 vs 36.9 months). For PD-1, RFS (hazard ratio [HR] 0.67) and OS (HR 0.66) were significantly longer in the high-expression group (45.2%). In the multivariate analysis, high TIM-3 expression (50.8%) had a significant relationship with shorter RFS (HR = 1.52) and OS (HR = 1.60). High CD3^+^ TIL and T-cell ICOS expression were associated with favourable prognosis, whereas high TIM-3 expression suggested a poor prognosis. Our findings may confer new insights to improve ESCC outcomes beyond the application of PD-1 blockade.

## Introduction

Oesophageal cancer is the sixth leading cause of global cancer-related death^1^. Despite developments in surgical and radiation techniques and chemotherapeutic agents, the prognosis of advanced oesophageal squamous cell carcinoma (ESCC) is generally poor, with a <35% 5-year survival rate^2^. There are two types of oesophageal cancer: ESCC and oesophageal adenocarcinoma. In Eastern Asia, >90% of oesophageal cancer cases are ESCC, with growing recognition that ESCC and oesophageal adenocarcinoma have distinct phenotypes, aetiologies, and pathogeneses^3^. The seventh and upcoming eighth edition cancer staging systems of the American Joint Committee on Cancer (AJCC) assign separate guidelines for staging these two cancer types^4^.

Immune escape occurs when cancer cells evade the host immune system. The interaction between programmed cell death ligand-1 (PD-L1) and programmed cell death protein-1 (PD-1) is a common and important immune-evading mechanism in many cancer types and blocking this interaction has emerged as a breakthrough in cancer therapy^5^. Since late 2016, PD-1 inhibitors have been approved for melanoma, non-small cell lung cancer, renal cell carcinoma, and head and neck cancer by the U.S. Food and Drug Administration; compared with old cytotoxic agents, PD-1 inhibitors have superior efficacy with an improved safety profile in various tumour types^6^. These checkpoint inhibitors have been adapted to daily clinical practice, dramatically changing malignant tumour treatment. Clinical trials with pembrolizumab and nivolumab are ongoing and include almost every tumour type from solid tumours to haematologic malignancies and from neoadjuvant and adjuvant to the palliative setting^7^.

Besides the PD-1/PD-L1 pathway, agents modulating other checkpoints, including inducible co-stimulator (ICOS), lymphocyte activation gene-3 (LAG-3), T-cell immunoglobulin and mucin-dominant containing-3 (TIM-3), and T-cell immunoreceptor with Ig and ITIM domains (TIGIT), are actively under development^8–10^. With PD-1/PD-L1 blockade as a backbone, combination approaches with these immune checkpoint modulators appear promising; this strategy may help overcome resistance to PD-1/PD-L1 inhibitors. Despite this approach, studies evaluating the prognostic and predictive impacts in each cancer type have produced inconsistent results^11,12^. The roles of PD-1, PD-L1, and other immune checkpoints in various cancer types remain unknown. Moreover, the clinical implications of various immune receptors in ESCC, including PD-1, PD-L1, LAG-3, TIM-3, and TIGIT, remain undefined.

Here, we investigated the distribution and frequency of CD3^+^/CD8^+^ T-cells, regulatory T-cells, and other immune checkpoints, including PD-L1, PD-1, ICOS, LAG-3, and TIM-3, in surgically resected ESCC and also determined their prognostic value in identifying potential therapeutic targets.

## Results

### Baseline characteristics

Overall, 407 patients who underwent surgical resection for ESCC were identified. Eleven cases were excluded due to incomplete staining, and 396 eligible ESCC tumour samples were assessed. They were evaluated for PD-L1 expression (on both tumour and immune cells) and CD3, CD8, Foxp3, ICOS, LAG-3, and TIM-3 expression (Supplementary Fig. 1). Table 1 shows the patients’ baseline characteristics. The median age was 64 years (range: 41–83 years); most patients were male (93.4%) and were current or former smokers (73.5%). The percentages of patients in stage I, II, III, and IV at surgery were 21.2%, 45.7%, 30.0%, and 3.0%, respectively. None of the patients received neoadjuvant treatment. During the median follow-up period of 24.8 (range 0.5–210) months, 129 (32.6%) patients experienced recurrence, and 181 (45.7%) deaths occurred. The 5-year overall survival (OS) rate was 44.9% in the total population.

**Table 1:** Baseline Characteristics in 396 ESCC Patients.

Characteristics	N (%)
**Age, years**	
Median (range)	64 (41–83)
**Sex**	
Male	370 (93.4%)
Female	26 (6.6%)
**Location**	
Upper	24 (6.1%)
Middle	84 (21.2%)
Lower	288 (72.7%)
**Tumor grade**	
Well	72 (18.2%)
Moderate	261 (65.9%)
Poorly	63 (15.9%)
**Smoking status**^**a**^	
Never smoker	103 (26.0%)
Former smoker	100 (25.2%)
Current smoker	193 (48.7%)
**Smoking dosage, pack-years**	
Median	25
Range	0–100
**pT stage**	
T1	119 (30.1%)
T2	81 (20.5%)
T3	181 (45.7%)
T4	15 (3.8%)
**pN stage**	
N0	202 (51.0%)
N1	165 (41.7%)
N2	20 (5.1%)
N3	9 (2.3%)
**pTNM stage (AJCC 7**^**th**^ **edition)**	
I	84 (21.2%)
II	181 (45.7%)
III	119 (30.0%)
IV	12 (3.0%)
**Adjuvant therapy**	
Yes	346 (87.4%)
No	50 (12.6%)

^a^Never smokers, a lifetime smoking dose of fewer than 100 cigarettes; former smokers, those who have stopped smoking for more than 1 year; current smokers, those who currently smoke or have quit for less than 1 year.

AJCC, American Joint Committee on Cancer; ESCC, oesophageal squamous cell carcinoma.

### Prognostic impact of CD3^+^, CD8^+^, and Foxp3^+^ T-cell lymphocyte density

**Figure 1:**
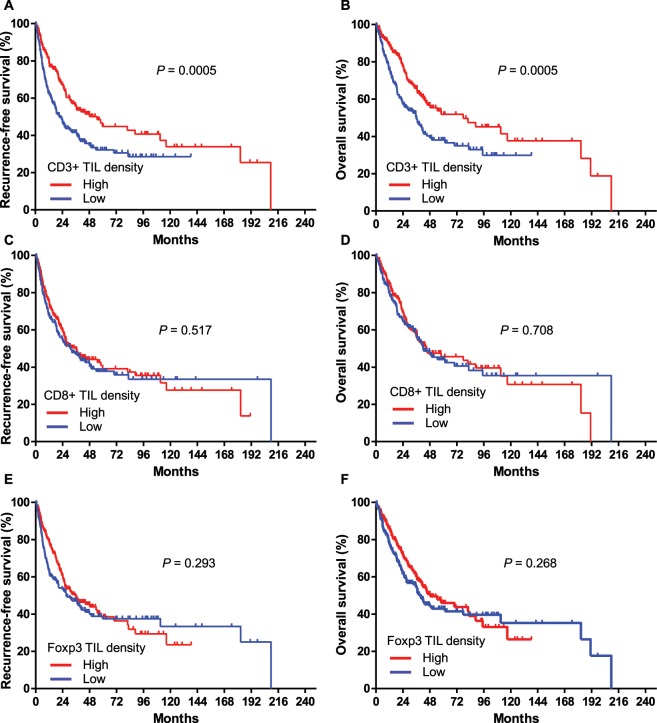
Kaplan-Meier analysis for probability of recurrence (**A,C,E**) and survival (**B,D,F**) based on the density of tumour-infiltrating lymphocytes. CD3 (**A,B**), CD8 (**C,D**), and Foxp3 (**E,F**).TIL, tumour-infiltrating lymphocyte.

Patients were grouped into low- and high-frequency groups based on tumour-infiltrating lymphocytes (TILs) according to a median value of 252/4HPFs for CD3^+^ TILs, 96/4HPFs for CD8^+^ TILs, and 0/4HPFs for Foxp3^+^ TILs. The association between the frequency of CD3^+^, CD8^+^, and Foxp3^+^ T-cell lymphocytes and the clinicopathological characteristics of patients are shown in Supplementary Tables 1–3, respectively. High densities of both CD3^+^ TIL and FoxP3 + TIL were associated with high pathologic T and TNM stage (Supplementary Tables 1 and 3). The high-frequency CD3^+^ TILs group (n = 198, 50.0%) had a significant longer RFS (hazard ratio [HR] = 0.61, 95% confidence interval [CI]: 0.46–0.81, *P* = 0.0005) and OS (HR = 0.59, 95% CI: 0.44–0.80, *P* = 0.0005) than the low frequency CD3^+^ TILs group (Fig. 1A,B). The high- and low-frequency CD3^+^ TILs groups had medians of 55.0 and 21.4 months for RFS and medians of 77.7 and 35.8 months for OS, respectively. After adjusting for age, sex, smoking status, and various T-cell marker expression levels, multivariate analysis demonstrated a low risk of disease relapse (HR = 0.57, 95% CI: 0.41–0.78, *P* < 0.001) and death (HR = 0.52, 95% CI: 0.37–0.74, *P* < 0.001) in the high-frequency CD3^+^ TILs group (Tables 2 and 3). However, there was no prognostic association between the density of CD8^+^ TILs or Foxp3^+^ TILs and RFS or OS (Fig. 1C–F).

**Table 2:** Uni- and Multivariate Cox Regression Analyses Estimating the Association of Various Immune Markers with Recurrence.

		Univariate analysis			Multivariate analysis		
	Category^a^	HR	95% CI	*P* value	HR	95% CI	*P* value
Age	≥60 or <60 (ref)	1.58	1.16–2.22	0.004	1.41	1.03–1.94	0.031
Sex	Female or male (ref)	0.57	0.30–1.07	0.055	0.64	0.32–1.28	0.211
Smoking	Ever-smoker or never-smoker (ref)	1.38	0.99–1.91	0.053	1.48	1.07–2.06	0.019
Tumor Location	Lower or middle or lower (ref)	1.21 1.06	0.77–1.91 0.73–1.52	0.771 0.411	1.25 1.05	0.78–1.98 0.75–1.66	0.778 0.795
Tumor Grade	Poorly or moderate or well (ref)	1.29 0.94	0.68–2.45 0.47–1.89	0.435 0.863	1.30 0.85	0.77–2.65 0.84–2.45	0.556 0.846
CD3^+^ T-cell	High or low (ref)	0.48	0.32–0.71	<0.001	0.57	0.41–0.78	<0.001
CD8^+^ T-cell	High or low (ref)	1.29	0.91–1.12	0.146	1.31	0.92–1.85	0.132
Foxp3^+^ T-cell	High or low (ref)	1.33	0.97–1.82	0.077	1.29	0.94–1.77	0.114
ICOS^+^ immune cell	High or low (ref)	0.62	0.45–0.85	0.003	0.67	0.50–0.89	0.007
LAG-3^+^ immune cell	High or low (ref)	0.98	0.70–1.38	0.910	0.95	0.67–1.35	0.777
PD-1^+^ immune cell	High or low (ref)	0.78	0.57–1.07	0.129	0.77	0.56–1.06	0.112
TIM-3^+^ Immune cell	High or low (ref)	1.44	1.02–2.03	0.040	1.52	1.10–2.10	0.011
PD-L1^+^ tumor cell (5% or more)	High or low (ref)	1.16	0.83–1.62	0.391	1.18	0.85–1.65	0.325
PD-L1^+^ immune cell (5% or more)	High or low (ref)	0.94	0.67–1.31	0.700	0.91	0.65–1.28	0.602
Stage III, IV vs I, II	High or low (ref)	3.26	2.42–4.37	<0.001	3.06	2.30–4.05	<0.001

Associations determined by Cox proportional hazards regression and adjusted for age, sex, smoking status, and TNM stage.

^a^Definition of high or low for each immune marker is described in the main manuscript.

ESCC, oesophageal squamous cell carcinoma.

**Table 3:** Uni- and Multivariate Cox Regression Analyses Estimating the Association of Various Immune Markers with Patients’ Survival.

		Univariate analysis			Multivariate analysis		
	Category^a^	HR	95% CI	*P* value	HR	95% CI	*P* value
Age	≥60 or <60 (ref)	1.66	1.18–2.31	0.003	1.47	1.05–2.06	0.026
Sex	Female or male (ref)	0.60	0.30–1.17	0.131	0.69	0.32–0.70	0.313
Smoking	Ever-smoker or never-smoker (ref)	1.32	0.94–1.86	0.110	1.44	1.01–2.04	0.042
Tumor Location	Lower or middle or lower (ref)	1.29 1.00	0.46–2.16 0.64–2.64	1.000 1.297	1.40 1.15	0.50–2.22 0.72–2.52	1.000 1.320
Tumor Grade	Poorly or moderate or well (ref)	1.15 1.04	0.71–1.87 0.70–1.53	0.58 0.86	1.10 0.98	0.70–1.89 0.68–1.55	0.69 0.85
CD3^+^ T-cell	High or low (ref)	0.48	0.32–0.71	<0.001	0.52	0.37–0.74	<0.001
CD8^+^ T-cell	High or low (ref)	1.38	0.97–1.99	0.078	1.38	0.96–1.98	0.087
Foxp3^+^ T-cell	High or low (ref)	1.29	0.93–1.79	0.133	1.29	0.93–1.80	0.132
ICOS^+^ immune cell	High or low (ref)	0.61	0.43–0.85	0.004	0.61	0.45–0.85	0.003
LAG-3^+^ immune cell	High or low (ref)	1.06	0.74–1.52	0.747	1.03	0.71–1.49	0.876
PD-1^+^ immune cell	High or low (ref)	0.81	0.58–1.14	0.220	0.80	0.57–1.12	0.192
TIM-3^+^ Immune cell	High or low (ref)	1.53	1.06–2.22	0.024	1.60	1.13–2.27	0.008
PD-L1^+^ tumor cell (5% or more)	High or low (ref)	0.96	0.68–1.41	0.891	1.00	0.69–1.44	0.987
PD-L1^+^ immune cell (5% or more)	High or low (ref)	0.76	0.52–1.10	0.146	0.73	0.50–1.07	0.106
Stage III, IV vs I, II	High or low (ref)	3.66	2.67–5.00	<0.001	3.34	2.47–4.51	<0.001

Associations determined by Cox proportional hazards regression and adjusted for age, sex, smoking status, and TNM stage.

^a^Definition of high or low for each immune marker is described in the main manuscript.

ESCC, oesophageal squamous cell carcinoma.

### Prognostic implication of ICOS, LAG-3, PD-1, and TIM-3 expression in immune cells

**Figure 2:**
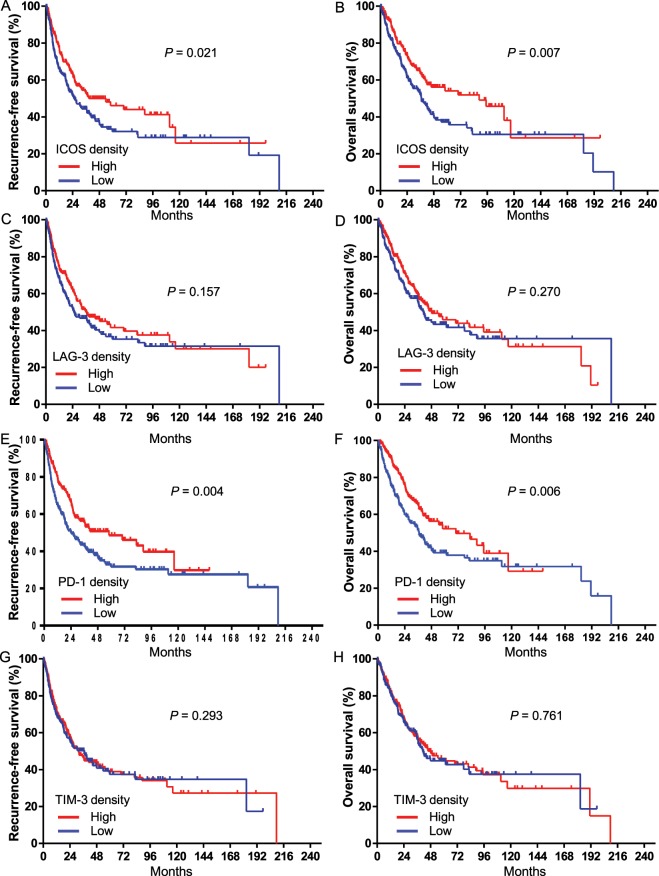
Kaplan-Meier analysis for probability of recurrence (**A,C,E,G**) and survival (**B,D,F,H**) based on the expression of each immune checkpoint. ICOS (**A,B**), LAG-3 (**C,D**), PD-1 (**E,F**), TIM-3 (**G,H**). ICOS, inducible co-stimulator; LAG-3, lymphocyte activation gene-3; PD-1, programmed cell death protein 1; TIM-3, T-cell immunoglobulin and mucin-dominant containing-3.

We investigated the expression of immune receptors (ICOS, LAG-3, PD-1, and TIM-3) in immune cells. The median values of ICOS, LAG-3, and PD-1 expression in immune cells were 0/4HPFs, 22/4HPFs, and 0/4HPFs, respectively. Given the abundance of TIM-3 expression, the proportion of TIM-3-positive immune cells was not numbered as the cell count/HPF but scored as the percentage of stained cells in the total immune cells. Because the median percentage of TIM-3 expression was 0%, TIM-3 expression of ≥1% was defined as high density. The association between the expression of ICOS, LAG-3, PD-1, and TIM-3 and clinicopathological characteristics of patients are shown in Supplementary Tables 4–7, respectively. Fisher’s exact test indicated that low ICOS expression was associated with smoking. Additionally, low pathologic T and pathologic TNM stage was related to high LAG-3 expression. There was no significant association between PD-1 expression and the clinical characteristics. In the survival analysis of these markers, patients with high ICOS expression (n = 184, 46.5%) demonstrated a significantly longer RFS (HR = 0.72, 95% CI: 0.55–0.95, *P = *0.021) and OS (HR = 0.67, 95% CI: 0.50–0.89, *P = *0.007) compared to that in patients with low ICOS expression (Fig. 2A,B). The high and low ICOS groups had medians of 53.9 and 25.3 months, respectively, for RFS and medians of 88.8 and 36.9 months, respectively, for OS. Similar results were observed in uni- and multivariate Cox analyses comparing high vs low ICOS expression (Tables 2 and 3; HR = 0.67, 95% CI: 0.50–0.89, *P* = 0.007 for recurrence; and HR = 0.61, 95% CI: 0.45–0.85, *P* = 0.003 for survival, all in multivariate analysis). LAG-3 expression was not associated with RFS and OS duration in either Kaplan-Meier or uni- and multivariate analyses (Fig. 2C,D, and Table 2). Regarding PD-1 expression, RFS (HR = 0.67, 95% CI: 0.50–0.88, *P* = 0.004) and OS (HR = 0.66, 95% CI: 0.49–0.88, *P* = 0.006) were significantly better in the high-expression group (n = 179) than in the low-expression group (n = 217) (Fig. 2E,F). While there was a trend toward better survival in patients with high PD-1 expression, Cox regression analysis indicated that associations between survival and PD-1 expression were non-significant (Tables 2 and 3). TIM-3 expression had no prognostic value for RFS and OS in the Kaplan-Meier analysis (Fig. 2G,H). However, in the uni- and multivariate analyses using Cox regression, high TIM-3 expression (n = 201, 50.8%) was associated with a higher probability of recurrence (HR = 1.52, 95% CI: 1.10–2.10, *P* = 0.011 in multivariate analysis) and death (HR = 1.60, 95% CI: 1.13–2.27, *P* = 0.008 in multivariate analysis) (Tables 2 and 3). To refer our finding to an independent patient cohort, we applied gene expression to The Cancer Genome Atlas (TCGA) ESCC samples regarding TIM-3 (n = 85, Supplementary Fig. 2). At first, mRNA expression data of hepatitis A virus cellular receptor 2 (*HAVCR2*) gene, which encodes the TIM-3 protein in ESCC, were obtained from the Genomic Data Commons Data Portal (CDC) in TCGA and OncoLnc. OncoLnc is a resource to link TCGA survival data to mRNA, miRNA, or lncRNA expression (available at http://www.oncolnc.org)^13,14^. There was no difference in OS between the high and low *HAVCR2* mRNA expressions (median OS, 42.1 and 18.9 months, respectively, HR = 0.67, 95% CI 0.3–1.48, *P* = 0.32).

### PD-L1 immunohistochemistry

Among 396 patients, 101 (25.5%), 89 (22.5%), 60 (15.7%), 33 (8.3%), and 14 (3.5%) had positive IHC staining of PD-L1 in ≥1%, ≥5%, ≥10%, ≥25%, and ≥50% of tumour cells, respectively. For immune cells, 189 (47.7%), 141 (35.6%), 97 (24.4%), 44 (11.1%), and 21 (5.3%) patients had ≥1%, ≥5%, ≥10%, ≥25%, and ≥50% of cells showing PD-L1 IHC staining, respectively. Seventy-four percent of tumour cells and 52% of immune cells did not express PD-L1 (Supplementary Fig. 3). With a 5% cut-off point, no significant correlation was observed between T-cell frequency and age, sex, tumour location, smoking status, and TNM stage. However, higher PD-L1 expression in tumour cells was related to a higher N stage (Supplementary Tables 8 and 9).

### Prognostic impact of PD-L1 expression on tumour and immune cells

**Figure 3:**
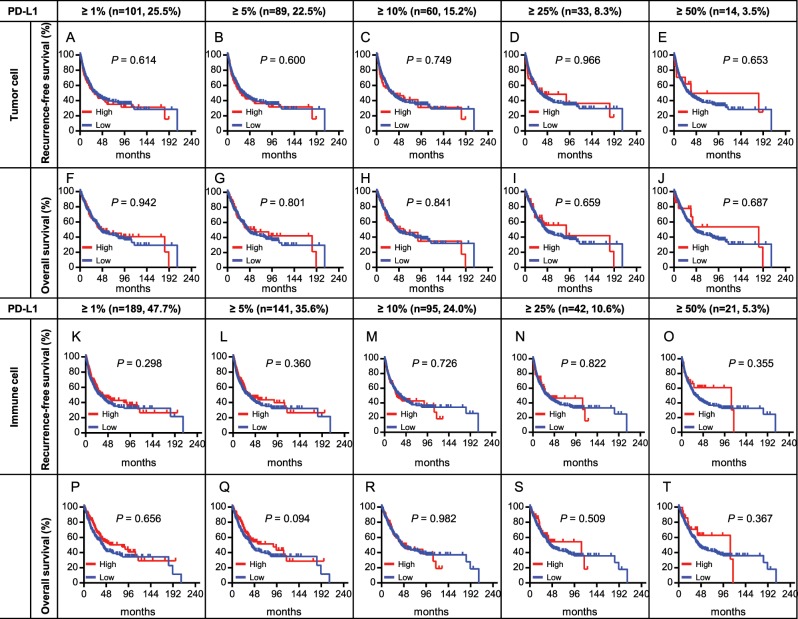
Kaplan-Meier plots showing the prognostic value of PD-L1 staining on tumour cell or immune cell, using different pre-specified cut-offs (1, 5, 10, 25, and 50%). (**A–J**) PD-L1 expression in tumour cell; (**K–T**) PD-L1 expression in immune cell; (**A–E**,**K–O**) recurrence-free survival; (**F–J**, **P–T**) overall survival.

PD-L1 expression on tumour and immune cells with various cut-offs (1%, 5%, 10%, 25%, and 50%) were evaluated among 396 samples and analysed in relation to the survival data (Fig. 3). The median PD-L1 expression rate was 0% in both tumour and immune cells, while the corresponding means were 5.6% and 8.0%, respectively. Even after incorporating various cut-off points in tumour and immune cells, our data indicated no significant correlation between PD-L1 expression and RFS and OS. Multivariate Cox analysis showed similar results (Tables 2 and 3). Median RFS and OS of patients with PD-L1 expression in ≥50% immune cells (n = 21, 5.3%) were numerically greater than those in patients with PD-L1 expression in <50% of cells (median RFS: 111.1 vs 30.7 months, *P* = 0.355, median OS: 111.1 vs 42.3 months, *P* = 0.367). However, this difference was not statistically significant.

### Relationships among expression of various immune markers

Overall, the median expression levels of FoxP3, ICOS, PD-1, and PD-L1 were 0, and any expression from these markers was considered as a “higher expression”. Moreover, the expression of one immune marker has close associations with other markers (Supplementary Table 10). After performing Spearman’s rank correlation analysis, CD8^+^ TIL expression was related with LAG-3, PD-1, TIM-3, and PD-L1 expression in immune cells (*P* < 0.001, respectively). Two immune markers, CD3 and ICOS, that displayed a prognostic role in this study were linked (*r* = 0.128, *P* = 0.011). However, no significant association was present between CD8 and ICOS expression or between ICOS and LAG-3 expression.

### Combination of CD3^+^ TIL with ICOS, PD-1, and TIM-3 expression

We classified ESCC subjects into four subgroups based on CD3^+^ TIL positivity and ICOS expression, and the same method was applied for CD3 with PD-1 and CD3 with TIM-3, as these were proposed to have a prognostic impact in the current analysis. The Kaplan-Meier analysis revealed significant differences in RFS among these four types in each subgroup (Supplementary Fig. 4). Consistent with the strong prognostic characteristics of ICOS and PD-1, CD3^+^ subgroups with positive ICOS or PD-1 expression showed the longest RFS (median: 88.8 and 58.0 months, respectively) compared with subgroups that were CD3^+^/ICOS- or CD3^+^/PD-1- (median: 18.6 and 17.6 months, respectively). Furthermore, consistent with the poor prognostic profile of TIM-3, subgroups that were CD3^+^/TIM-3^−^ demonstrated the longest RFS (median: 59.7 vs 3.5 months in CD3^−^/TIM-3^−^). However, these correlations with RFS did not translate into OS.

## Discussion

ESCC is one of the most aggressive cancers. Definitive surgical therapy, including oesophagectomy, is considered a standard treatment for resectable diseases; however, >50% of patients undergo local recurrence and/or distant metastasis^15,16^. Even with recent improvements in sequencing techniques and precision medicine, molecularly targeted therapy has a limited role. Furthermore, traditional cytotoxic agents have not demonstrated progress in the last decade. Therefore, ESCC prognosis is dismal, with a high unmet need. In other cancer types such as lung cancer, immune checkpoint inhibitors, represented by PD-1/PD-L1 blockade, have recently shown promising efficacy in treating advanced/metastatic ESCC^17,18^. In a clinical trial, the objective response rate of 17% and a median OS of 10.8 months appear to be encouraging considering the heavily treated characteristics in the study population^18^. Despite the impressive outcomes of PD-1/PD-L1 inhibitory cancer immunotherapy, efficacy is suboptimal, and most patients with many tumour types do not show a response^19^. Thus, the focus has shifted to targeting alternative immune checkpoints receptors.

Combining two or more anti-tumour drugs for improving cancer treatment is not a new concept in oncology, and it is also valid in the immuno-oncology era^20^. Combination therapy involving PD-1/PD-L1 inhibitors with other immune checkpoint modulators has been considered an important strategy to overcome primary and secondary resistances to PD-1/PD-L1 blockade^21^. Several immune modulators are being investigated in pre-clinical models and clinical trials; however, our understanding of these checkpoints is incomplete, especially in ESCC. Furthermore, the expression level cut-offs, associations with clinicopathological features, and prognostic impact of these various checkpoints have not been established for ESCC. The PD-L1 expression level has a critical role in T-cell regulation, but the precise significance varies among studies and cancer types^22^.

We comprehensively investigated the prognostic impact of immune cell infiltration and various immune checkpoints, including PD-L1, PD-1, ICOS, LAG-3, and TIM-3, in resected ESCC. Additionally, the significance of clinical factors such as sex, age, smoking status, and TNM stage was explored. The most significant prognostic factor was the TNM stage (Table 2 and Supplementary Fig. 5). Given that the number of stage IV subjects was small, it may not have a significant impact on OS and mitigate the influence of immune markers.

Several studies report that TILs have a positive prognostic effect across various cancer types^23–25^. TILs have an important role in anticancer immune responses and the cancer immunity cycle^26^. However, TILs are divided into several subgroups based on cell surface antigen expression. Each subset has a distinct interactive function between the tumour and tumour microenvironments and sometimes the roles differ across cancer types^27^. CD3 is a transmembrane protein that is exclusively expressed in the T-cell pedigree and thus is considered to represent a mature T-cell marker and whole tumour-infiltrating T-cells^28^. CD8 T-cells generally represent cytotoxic T-cells, and Foxp3 is the distinct transcription marker of regulatory T-cells^29^. Among different lymphocyte types, CD3-positive TILs are most significantly associated with survival across various tumours^27^. Consistent with previous literature on total TILs or stromal TILs in ESCC, in the present ESCC study, high CD3^+^ TIL density showed good prognostic impact, whereas CD8^+^ TILs and Foxp3^+^ TILs did not^30–32^. We attempted to investigate stromal and intra-tumoural CD3^+^ or CD8^+^ TILs independently; however, TIL abundance made it unfeasible. One meta-analysis showed that CD8^+^ TILs were associated with good prognosis; however, it included a heterogeneous population of patients with oesophageal adenocarcinoma and squamous cell carcinoma^33^.

To our knowledge, we are the first to report ICOS, LAG-3, and TIM-3 expressions and their clinical implication in a large ESCC cohort. As expected, ICOS, LAG-3, and TIM-3 expressions were related to PD-1 expression on TILs, possibly indicating the feedback nature of the immune system (Supplementary Table 10).

ICOS is a surface antigen in T-cells, and its expression is low in naïve T-cells. Once ICOS is stimulated by both the T-cell receptor and CD28 signals, it is upregulated in activated T-cells^34^. Co-stimulation by ICOS and ICOS ligand confers an anticancer response, but ICOS signalling also engages regulatory T-cell activity induction^35^. Thus, ICOS might have a dual role in oncogenesis. The prognostic impact of ICOS expression is inconsistent across various tumour types (e.g., melanoma, colorectal cancer, breast cancer, gastric cancer, and renal cell carcinoma), and these clinical findings support the dual role of ICOS in carcinogenesis^8^. In our study, high ICOS expression in immune cells was significantly associated with longer RFS and OS in the Kaplan-Meier method and multivariate Cox regression model (Fig. 2 and Table 2). Our findings suggest that ICOS-expressing immune cells include tumour neoantigen-specific T-cells and are associated with favourable prognosis, at least in ESCC. However, this should be validated in an independent patient cohort and in clinical trials with ICOS-targeted therapies.

Despite the existence of several LAG-3-related studies, data regarding the prognostic impact of LAG-3 in most cancer types are limited^9^. Two reports on NSCLC have contradictory results^36,37^. Moreover, one exploratory study showed that LAG-3 expression was associated with good prognosis in resected ESCC; however, this is inconsistent with the current data^38^. Accordingly, the role of LAG-3 in cancer and ESCC in particular remains elusive.

As a marker of CD8^+^ T-cell dysfunction or exhaustion, TIM-3 is occasionally co-expressed with PD-1 in CD8^+^ T-cells in several tumours^19^. Moreover, TIM-3 is expressed in natural killer cells, regulatory T-cells, dendritic cells, B-cells, macrophages, and other myeloid cells. TIM-3 expression inhibited anti-tumour immunity by T-cell exhaustion, suppressed immune response from the status of innate immunity, and exhibited pro-tumour activity by promoting myeloid-derived suppressor cells^9^. Consistent with the previously known immune regulatory role of TIM-3 in oncogenesis, TIM-3 expression is associated with poor prognosis in several cancers^39,40^. The high probability of recurrence or death was also related to high TIM-3 expression in the present study, which is the first to report the prognostic impact of TIM-3 in ESCC. In several preclinical studies, blocking TIM-3 enhanced cancer immunity by T-cell proliferation and cytokine production, as well as combination with PD-1 blockade, also demonstrated a remarkable synergy in these models^10^. Additionally, many clinical trials focusing on targeting TIM-3 are ongoing globally^41^. One important issue regarding blocking TIM-3 might be related to their expression levels in various tumour-infiltrating immune cells. Most patients (92%, 364/396, data not shown) in the present study expressed TIM-3 in T-cells. Thus, based on the high frequency of TIM-3 expression and its demonstrated poor prognostic value, TIM-3 may be a potential target candidate in ESCC. In conjunction with the current role of PD-1 blocking agents in ESCC, the use of TIM-3-targeted therapy in combination with PD-1 blockade may be a promising strategy. Although more studies are needed, our study may provide some evidence supporting the approach to target TIM-3 in ESCC.

Together with advances in PD-1/PD-L1 blockade, the relationship of PD-1/PD-L1 expression and its prognostic impact in various tumour types are gaining increased attention. Although the Kaplan-Meier method revealed that high PD-1 expression in TILs was associated with better RFS and OS, in Cox regression analyses, patients with high PD-1 expression had increased risk of relapse and death compared with those with low PD-1 expression. Considering the high association with PD-1 and other immune checkpoints, the overlap and diverse interaction between various immune checkpoints may attenuate the prognostic impact of PD-1 in ESCC (Supplementary Table 10).

Regarding PD-L1, 25.5% (101/396) of patients showed PD-L1 expression in ≥1% of tumour cells and in 47.7% (189/396) of immune cells (Supplementary Fig. 3). We used various cut-offs of PD-L1 expression in tumour or immune cells to determine associations with prognosis, but we did not identify any meaningful associations with recurrence or survival. Because blocking PD-1/PD-L1 has gained much attention, several studies have investigated the prognostic role of PD-L1 expression in ESCC. However, these studies presented inconsistent results^30^. Some studies reported that PD-L1 expression was associated with poor prognosis, but another study indicated it was not associated with poor prognosis. A meta-analysis showed a positive but statistically non-significant trend in prognosis with PD-L1 expression^42^. In our study, there were no clinically significant relationships between survival and PD-L1 expression in tumour or immune cells. These conflicting results might derive from differences in the patient population, staining method and antibodies, and definition of PD-L1 positivity. Thus, heterogeneity in the prognostic role of PD-L1 has been observed in several cancer types.

Our study has some limitations. First, it was retrospective in nature. Second, it did not include the localisation information of each marker and therefore did not provide data on the spatial distribution of immune cells. Furthermore, since most patients underwent oesophagectomy when the neoadjuvant concurrent chemoradiation data was published and incorporated into clinical practice, none of the patients received preoperative treatment^43^. Additionally, no patient underwent immunotherapy; thus, this study did not provide any data regarding the response to immune checkpoint modulators.

In conclusion, we demonstrated that a high frequency of CD3^+^ TILs and high ICOS expression on immune cells are related to a favourable prognosis in ESCC. Furthermore, TIM-3 expression in immune cells was associated with poor prognosis in uni- and multivariate analyses. Our findings may help improve ESCC outcomes beyond the application of PD-1 blockade. These discoveries have important implications for using antibody therapies in addition to the PD-1/PD-L1 signalling pathway, such as TIM-3, in ESCC.

## Methods

### Patients and tissue samples

The study population comprised ESCC patients who underwent radical surgical resection at Severance Hospital and Samsung Medical Center, Seoul, Korea between 1996 and 2012. The inclusion criteria were (1) surgically resected ESCC with a curative aim and (2) availability of tumour tissue. Clinicopathologic data were collected and reviewed. Tumours were re-classified based on the 7^th^ edition of the AJCC TNM cancer staging system. Twelve subjects underwent oesophagectomy and were subsequently identified as having stage IV. The study was approved by the Institutional Review Board (IRB) of Severance Hospital and was conducted according to the ethical principles, guidelines, and relevant regulations. The IRB approved our research, and as the study was a retrospective review of subjects, the requirement for informed consent was waived.

### Tissue microarray preparation and IHC

ESCC specimens were histologically reviewed by two experienced pathologists (Y.C. and S.S.). Haematoxylin- and eosin-stained sections from formalin-fixed paraffin-embedded tissues were reviewed to identify the invasive carcinoma area. The most densely viable carcinoma areas were chosen as the representative areas (core – 3.0 mm). Tissue microarrays were constructed from these samples and assessed by immunohistochemical staining for PD-L1, PD-1, CD3, CD8, Foxp3 (forkhead box P3), ICOS, TIM-3, and LAG-3.

The following primary antibodies were used: PD-L1 (dilution 1:100; clone SP142; Ventana), PD-1 (dilution 1:100; clone NAT105; Cell Marque, Rocklin, CA, USA), CD3 (dilution 1:200; LabVision, Fremont, CA, USA), CD8 (RTU; clone C8/144B; Dako, Glostrup, Denmark), Foxp3 (dilution 1:100; Abcam, Cambridge, UK), ICOS (clone SP98; dilution 1:50; Thermo Scientific, Rockford, IL, USA), TIM-3 (dilution 1:200; clone D5D5R^TM^; Cell Signaling), and LAG-3 (clone EPR4392(2); dilution 1:100; Abcam). The staining protocols were the same as published previously^44^.

### IHC scoring

Two well-experienced pathologists manually scored each IHC in the tissue microarray samples. PD-L1 expression on tumour and immune cells was analysed separately. Tumour cells showing any intensity of membranous and/or cytoplasmic staining were defined as having positive staining, and the proportion of PD-L1-positive tumour cells was estimated as the percentage of total tumour cells in the whole section. The pre-specified cut-off values at 1%, 5%, 10%, 10%, 25%, and 50% were established. The same method was applied to assess PD-L1 expression in immune cells. The expression was defined as high density for CD3, CD8, ICOS, Foxp3, LAG-3, and PD-1 when the expression level was above the median value per four high-power fields (4HPFs).

### Statistical analysis

We investigated the association between various immune markers and clinical data using the chi-squared test or Fisher’s exact test. Survival was analysed by the Kaplan-Meier method with log-rank for categorical variables. Uni- and multivariate analyses for predicting recurrence and survival were performed using the Cox regression method for the following variables: age, sex, smoking status, stage, histology, location, and each immune marker status. RFS was defined from the time of surgery to initial relapse or death. OS was defined as the time from the surgery until death from any cause or the most recent follow-up. The cases for OS included cases that were still alive and cases whose causes of death were not related to oesophageal cancer. Living patients were censored at the time of the last follow-up. Spearman’s rank correlation test was used to assess the relationship among various immune markers. All statistical analyses were performed using SPSS version 23.0 (SPSS, Chicago, IL). *P* < 0.05 was considered statistically significant by the Kaplan-Meier method.

## Supplementary information

Supplementary information

## Data Availability

As per institutional policy, the datasets generated and analysed during the current study are publicly unavailable; however, they are available from the corresponding author upon reasonable request.
