# Supplementary material for: High CD3 and ICOS and low TIM-3 expression predict favourable survival in resected oesophageal squamous cell carcinoma
Source: Sci Rep. 2019 Dec 27;9:20197. doi: 10.1038/s41598-019-56828-7 (PMC6934772; doi:10.1038/s41598-019-56828-7)
Supplement: Supplementary file 1 — Supplementary information [file 41598_2019_56828_MOESM1_ESM.pdf]

Supplementary Files

High CD3 and ICOS and low TIM-3 expression predict favourable survival in resected oesophageal squamous cell carcinoma

Min Hee Hong[1], Su-Jin Shin[2], Sung Kwan Shin[3], Dae Joon Kim[4], Jae Ill Zo[5], Young Mog Shim[5], Seung Eun Lee[6], Byoung Chul Cho[1], Seong Yong Park[4], Yoon-La Choi[7,*] & Hye Ryun Kim[1,*]

[1]Division of Medical Oncology, Department of Internal Medicine, Yonsei Cancer Center, Severance Hospital, Yonsei University College of Medicine, Seoul, Korea

[2]Department of Pathology, Gangnam Severance Hospital, Yonsei University College of Medicine, Seoul, Korea

[3]Division of Gastroenterology, Department of Internal Medicine, Yonsei University College of Medicine, Seoul, Korea

[4]Department of Thoracic and Cardiovascular Surgery, Yonsei University College of Medicine, Seoul, Korea

[5]Department of Thoracic and Cardiovascular Surgery, Samsung Medical Center, Sungkyunkwan University School of Medicine, Seoul, Korea

[6]Department of Pathology, Konkuk University Medical Center, Konkuk University School of Medicine, Seoul, Republic of Korea

[7]Department of Pathology and Translational Genomics, Samsung Medical Center, Sungkyunkwan University School of Medicine, Seoul, Korea

Supplementary Files

Supplementary Table (Number 10)

Supplementary Figure (Number 5)

Supplementary Table 1. Association of $CD3^{+}$ T-cells with Clinicopathological Factors in ESCC

		$CD3^{+}$ T-cells[b]		*P* value
Characteristics	All cases	High	Low	
Total	396	198 (50%)	198 (50%)	
Age, years				0.67
<60	126 (31.8%)	65 (51.6%)	61 (48.4%)	
≥60	270 (68.2%)	133 (49.3%)	137 (50.7%)	
Sex				0.69
Male	370 (93.4%)	184 (49.7%)	186 (50.3%)	
Female	26 (6.6%)	14 (53.8%)	12 (46.2%)	
Location				0.87
Upper	24 (6.1%)	11 (45.8%)	13 (54.2%)	
Middle	84 (21.2%)	41 (48.8%)	43 (51.2%)	
Lower	288 (72.7%)	146 (50.7%)	142 (49.3%)	
Tumour grade				0.10
Well	72 (18.2%)	29 (40.3%)	43 (59.7%)	
Moderate	261 (65.9%)	132 (50.6%)	129 (49.4%)	
Poorly	63 (15.9%)	37 (58.7%)	26 (41.3%)	
Smoking status[a]				0.63
Never smoker	103 (26.0%)	49 (47.6%)	54 (52.4%)	
Former smoker	100 (24.7%)	54 (54.0%)	46 (46.0%)	
Current smoker	193 (48.7%)	95 (49.2%)	98 (50.8%)	
pT stage				< 0.001
T1	119 (30.1%)	83 (69.7%)	36 (30.3%)	
T2	81 (20.5%)	41 (50.6%)	40 (49.4%)	
T3	181 (45.7%)	68 (37.6%)	113 (62.4%)	
T4	15 (3.8%)	6 (40.0%)	9 (60.0%)	
pN stage				0.75
N0	202 (51.0%)	102 (50.5%)	100 (49.5%)	
N1	165 (41.7%)	79 (47.9%)	86 (52.1%)	
N2	20 (5.1%)	12 (60.0%)	8 (40.0%)	
N3	9 (2.3%)	5 (55.6%)	4 (44.4%)	
pTNM stage				0.003
I	84 (21.2%)	55 (65.5%)	29 (34.5%)	
II	181 (45.7%)	90 (49.7%)	91 (50.3%)	
III	119 (30.0%)	50 (42.0%)	69 (58.0%)	
IV	12 (3.0%)	3 (25.0%)	9 (75.0%)	

ICOS, inducible co-stimulator; ESCC, oesophageal squamous cell carcinoma.

[a]Never smokers, a lifetime smoking dose of fewer than 100 cigarettes; former smokers, those who have stopped smoking for more than 1 year; current smokers, those who currently smoke or have quit for less than 1 year.

[b]The high frequency of $CD3^{+}$ tumour-infiltrating lymphocytes is defined in the main manuscript.

Supplementary Table 2. Association of CD8+ T-Cells with Clinicopathological Factors in ESCC

		CD8+ T-cells[b]		*P* value
Characteristics	All cases	High	Low	
Total	396	202 (51.0%)	194 (49.0%)	
Age, years				0.18
<60	126 (31.8%)	58 (46.0%)	68 (54.0%)	
≥60	270 (68.2%)	144 (53.3%)	126 (46.7%)	
Sex				0.13
Male	370 (93.4%)	185 (50.0%)	185 (50.0%)	
Female	26 (6.6%)	17 (65.4%)	9 (34.6%)	
Location				0.37
Upper	24 (6.1%)	9 (37.5%)	15 (62.5%)	
Middle	84 (21.2%)	45 (53.6%)	39 (46.4%)	
Lower	288 (72.7%)	148 (51.4%)	140 (48.6%)	
Tumour grade				0.25
Well	72 (18.2%)	32 (44.4%)	40 (55.6%)	
Moderate	261 (65.9%)	133 (51.0%)	128 (49.0%)	
Poorly	63 (15.9%)	37 (58.7%)	26 (41.3%)	
Smoking status[a]				0.57
Never smoker	103 (26.0%)	56 (54.4%)	47 (45.6%)	
Former smoker	100 (24.7%)	47 (47.0%)	53 (53.0%)	
Current smoker	193 (48.7%)	99 (51.3%)	94 (48.7%)	
pT stage				0.19
T1	119 (30.1%)	69 (58.0%)	50 (42.0%)	
T2	81 (20.5%)	43 (53.1%)	38 (46.9%)	
T3	181 (45.7%)	84 (46.4%)	97 (53.6%)	
T4	15 (3.8%)	6 (40.0%)	9 (60.0%)	
pN stage				0.85
N0	202 (51.0%)	101 (50.0%)	101 (50.0%)	
N1	165 (41.7%)	84 (50.9%)	81 (49.1%)	
N2	20 (5.1%)	12 (60.0%)	8 (40.0%)	
N3	9 (2.3%)	5 (55.6%)	4 (44.4%)	
pTNM stage				0.56
I	84 (21.2%)	47 (56.0%)	37 (44.0%)	
II	181 (45.7%)	94 (51.9%)	87 (48.1%)	
III	119 (30.0%)	56 (47.1%)	63 (52.9%)	
IV	12 (3.0%)	5 (41.7%)	7 (58.3%)	

ICOS, inducible co-stimulator; ESCC, oesophageal squamous cell carcinoma.

[a]Never smokers, a lifetime smoking dose of fewer than 100 cigarettes; former smokers, those who have stopped smoking for more than 1 year; current smokers, those who currently smoke or have quit for less than 1 year.

[b]The high frequency of CD8+ tumour-infiltrating lymphocytes is defined in the main manuscript.

Supplementary Table 3. Association of FoxP3[+] T-Cells with Clinicopathological Factors in ESCC

		FoxP3[+] T-cells		*P* value
Characteristics	All cases	High	Low	
Total	396	211 (53.3%)	185 (46.7%)	
Age, years				0.54
<60	126 (31.8%)	70 (55.6%)	56 (44.4%)	
≥60	270 (68.2%)	141 (52.2%)	129 (47.8%)	
Sex				0.95
Male	370 (93.4%)	197 (53.2%)	173 (46.8%)	
Female	26 (6.6%)	14 (53.8%)	12 (46.2%)	
Location				0.16
Upper	24 (6.1%)	13 (54.2%)	11 (45.8%)	
Middle	84 (21.2%)	37 (44.0%)	47 (56.0%)	
Lower	288 (72.7%)	161 (55.9%)	127 (44.1%)	
Tumour grade				0.40
Well	72 (18.2%)	34 (47.2%)	38 (52.8%)	
Moderate	261 (65.9%)	140 (53.6%)	121 (46.4%)	
Poorly	63 (15.9%)	37 (58.7%)	26 (41.3%)	
Smoking status[a]				< 0.001
Never smoker	103 (26.0%)	47 (45.6%)	56 (54.4%)	
Former smoker	100 (24.7%)	72 (72.0%)	28 (28.0%)	
Current smoker	193 (48.7%)	92 (47.7%)	101 (52.3%)	
pT stage				< 0.001
T1	119 (30.1%)	95 (79.8%)	24 (20.2%)	
T2	81 (20.5%)	42 (51.9%)	39 (48.1%)	
T3	181 (45.7%)	69 (38.1%)	112 (61.9%)	
T4	15 (3.8%)	5 (33.3%)	10 (66.7%)	
pN stage				0.02
N0	202 (51.0%)	119 (58.9%)	83 (41.1%)	
N1	165 (41.7%)	73 (44.2%)	92 (55.8%)	
N2	20 (5.1%)	14 (70.0%)	6 (30.0%)	
N3	9 (2.3%)	5 (55.6%)	4 (44.4%)	
pTNM stage				< 0.001
I	84 (21.2%)	67 (79.8%)	17 (20.2%)	
II	181 (45.7%)	94 (51.9%)	87 (48.1%)	
III	119 (30.0%)	47 (39.5%)	72 (60.5%)	
IV	12 (3.0%)	3 (25.0%)	9 (75.0%)	

ICOS, inducible co-stimulator; ESCC, oesophageal squamous cell carcinoma.

[a]Never smokers, a lifetime smoking dose of fewer than 100 cigarettes; former smokers, those who have stopped smoking for more than 1 year; current smokers, those who currently smoke or have quit for less than 1 year.

[b]The high frequency of FoxP3[+] tumour-infiltrating lymphocytes is defined in the main manuscript.

Supplementary Table 4. Association of ICOS Expression in T-Cells with Clinicopathological Factors in ESCC

		ICOS Expression[b]		*P* value
Characteristics	All cases	High	Low	
Total	396	184 (46.5%)	212 (53.5%)	
Age, years				0.163
<60	126 (31.8%)	65 (51.6%)	61 (48.4%)	
≥60	270 (68.2%)	119 (44.1%)	151 (55.9%)	
Sex				0.974
Male	370 (93.4%)	172 (46.5%)	198 (53.5%)	
Female	26 (6.6%)	12 (46.2%)	14 (53.8%)	
Location				0.812
Upper	24 (6.1%)	12 (50.0%)	12 (50.0%)	
Middle	84 (21.2%)	41 (48.8%)	43 (51.2%)	
Lower	288 (72.7%)	131 (45.5%)	157 (54.5%)	
Tumour grade				0.107
Well	72 (18.2%)	27 (37.5%)	45 (62.5%)	
Moderate	261 (65.9%)	131 (50.2%)	130 (49.8%)	
Poorly	63 (15.9%)	26 (41.3%)	37 (58.7%)	
Smoking status[a]				0.003
Never smoker	103 (26.0%)	46 (44.7%)	57 (55.3%)	
Former smoker	100 (24.7%)	61 (61.0%)	39 (39.0%)	
Current smoker	193 (48.7%)	77 (39.9%)	116 (60.1%)	
pT stage				0.229
T1	119 (30.1%)	61 (51.3%)	58 (48.7%)	
T2	81 (20.5%)	40 (49.4%)	41 (50.6%)	
T3	181 (45.7%)	79 (43.6%)	102 (56.4%)	
T4	15 (3.8%)	4 (26.7%)	11 (73.3%)	
pN stage				0.032
N0	202 (51.0%)	91 (45.0%)	111 (55.0%)	
N1	165 (41.7%)	76 (46.1%)	89 (53.9%)	
N2	20 (5.1%)	15 (75.0%)	5 (25.0%)	
N3	9 (2.3%)	2 (22.2%)	7 (77.8%)	
pTNM stage				0.376
I	84 (21.2%)	45 (53.6%)	39 (46.4%)	
II	181 (45.7%)	79 (43.6%)	102 (56.4%)	
III	119 (30.0%)	56 (47.1%)	63 (52.9%)	
IV	12 (3.0%)	4 (33.3%)	8 (66.7%)	

ICOS, inducible co-stimulator; ESCC, oesophageal squamous cell carcinoma.

[a]Never smokers, a lifetime smoking dose of fewer than 100 cigarettes; former smokers, those who have stopped smoking for more than 1 year; current smokers, those who currently smoke or have quit for less than 1 year.

[b]The high expression of ICOS is defined in the main manuscript.

Supplementary Table 5. Association of LAG-3 Expression in T-cells with Clinicopathological Factors in ESCC

		LAG-3 Expression[b]		*P* value
Characteristics	All cases	High	Low	
Total	396	199 (50.3%)	197 (49.7%)	
Age, years				0.114
<60	126 (31.8%)	56 (44.4%)	70 (55.6%)	
≥60	270 (68.2%)	143 (53.0%)	127 (47.0%)	
Sex				0.665
Male	370 (93.4%)	187 (50.5%)	183 (49.5%)	
Female	26 (6.6%)	12 (46.2%)	14 (53.8%)	
Location				0.249
Upper	24 (6.1%)	16 (66.7%)	8 (33.3%)	
Middle	84 (21.2%)	42 (50.0%)	42 (50.0%)	
Lower	288 (72.7%)	141 (49.0%)	147 (51.0%)	
Tumour grade				0.042
Well	72 (18.2%)	28 (38.9%)	44 (61.1%)	
Moderate	261 (65.9%)	133 (51.0%)	128 (49.0%)	
Poorly	63 (15.9%)	38 (60.3%)	25 (39.7%)	
Smoking status[a]				0.037
Never smoker	103 (26.0%)	51 (50.5%)	52 (49.5%)	
Former smoker	100 (24.7%)	61 (61.0%)	39 (39.0%)	
Current smoker	193 (48.7%)	87 (45.1%)	106 (54.9%)	
pT stage				0.001
T1	119 (30.1%)	75 (63.0%)	44 (37.0%)	
T2	81 (20.5%)	43 (53.1%)	38 (46.9%)	
T3	181 (45.7%)	77 (42.5%)	104 (57.5%)	
T4	15 (3.8%)	4 (26.7%)	11 (73.3%)	
pN stage				0.356
N0	202 (51.0%)	105 (52.0%)	97 (48.0%)	
N1	165 (41.7%)	76 (46.1%)	89 (53.9%)	
N2	20 (5.1%)	13 (65.0%)	7 (35.0%)	
N3	9 (2.3%)	5 (55.6%)	4 (44.4%)	
pTNM stage				0.002
I	84 (21.2%)	57 (67.9%)	27 (32.1%)	
II	181 (45.7%)	86 (47.5%)	95 (52.5%)	
III	119 (30.0%)	52 (43.7%)	67 (56.3%)	
IV	12 (3.0%)	4 (33.3%)	8 (66.7%)	

LAG-3, lymphocyte activation gene-3; ESCC, oesophageal squamous cell carcinoma.

[a]Never smokers, a lifetime smoking dose of fewer than 100 cigarettes; former smokers, those who have stopped smoking for more than 1 year; current smokers, those who currently smoke or have quit for less than 1 year.

[b]The high expression of LAG-3 is defined in the main manuscript.

Supplementary Table 6. Association of PD-1 Expression in Immune Cell with Clinicopathological Factors in ESCC

		PD-1 Immune Cells (5%)[b]		*P* value
Characteristics	All cases	Positive	Negative	
	396	189 (52.3%)	189 (47.7%)	
Age, years				0.372
<60	126 (31.8%)	56 (44.4%)	70 (55.6%)	
≥60	270 (68.2%)	133 (49.3%)	137 (50.7%)	
Sex				0.166
Male	370 (93.4%)	180 (48.6%)	190 (51.4%)	
Female	26 (6.6%)	9 (34.6%)	17 (65.4%)	
Location				0.737
Upper	24 (6.1%)	13 (54.2%)	11 (45.8%)	
Middle	84 (21.2%)	38 (45.2%)	46 (54.8%)	
Lower	288 (72.7%)	138 (47.9%)	150 (52.1%)	
Tumour grade				0.256
Well	72 (18.2%)	29 (40.3%)	54 (59.7%)	
Moderate	261 (65.9%)	132 (50.6%)	129 (49.4%)	
Poorly	63 (15.9%)	28 (44.4%)	35 (55.6%)	
Smoking status[a]				0.146
Never smoker	103 (26.0%)	48 (46.9%)	55 (53.4%)	
Former smoker	100 (24.7%)	56 (56.0%)	44 (44.0%)	
Current smoker	193 (48.7%)	85 (44.0%)	108 (56.0%)	
pT stage				0.163
T1	119 (30.1%)	62 (52.1%)	57 (47.9%)	
T2	81 (20.5%)	44 (54.3%)	37 (45.7%)	
T3	181 (45.7%)	78 (43.1%)	103 (56.9%)	
T4	15 (3.8%)	5 (33.3%)	10 (66.7%)	
pN stage				0.221
N0	202 (51.0%)	92 (45.5%)	110 (54.5%)	
N1	165 (41.7%)	79 (47.9%)	86 (52.1%)	
N2	20 (5.1%)	14 (70.0%)	6 (30.0%)	
N3	9 (2.3%)	4 (44.4%)	5 (55.6%)	
pTNM stage				0.393
I	84 (21.2%)	47 (56.%)	37 (44.0%)	
II	181 (45.7%)	82 (45.3%)	99 (54.7%)	
III	119 (30.0%)	54 (45.4%)	65 (54.6%)	
IV	12 (3.0%)	6 (50.0%)	6 (50.0%)	

PD-1, programmed cell death-1; ESCC, oesophageal squamous cell carcinoma.

[a]Never smokers, a lifetime smoking dose of fewer than 100 cigarettes; former smokers, those who have stopped smoking for more than 1 year; current smokers, those who currently smoke or have quit for less than 1 year.

[b]The positivity of PD-1 expression is defined in the main manuscript.

Supplementary Table 7. Association of TIM-3 Expression in Immune Cell with Clinicopathological Factors in ESCC

		TIM-3 Expression[b]		*P* value
Characteristics	All cases	High	Low	
Total	396	201 (50.8%)	195 (49.2%)	
Age, years				0.992
<60	126 (31.8%)	64 (50.8%)	62 (49.2%)	
≥60	270 (68.2%)	137 (50.7%)	133 (49.3%)	
Sex				0.464
Male	370 (93.4%)	186 (50.3%)	184 (49.7%)	
Female	26 (6.6%)	15 (57.7%)	11 (42.3%)	
Location				0.658
Upper	24 (6.1%)	12 (50.0%)	12 (50.0%)	
Middle	84 (21.2%)	39 (46.4%)	45 (53.6%)	
Lower	288 (72.7%)	150 (52.1%)	138 (47.9%)	
Tumour grade				0.219
Well	72 (18.2%)	30 (41.7%)	42 (58.3%)	
Moderate	261 (65.9%)	139 (53.3%)	122 (46.7%)	
Poorly	63 (15.9%)	32 (50.8%)	31 (49.2%)	
Smoking status[a]				0.077
Never smoker	103 (26.0%)	56 (54.4%)	47 (45.6%)	
Former smoker	100 (24.7%)	58 (58.0%)	42 (42.0%)	
Current smoker	193 (48.7%)	87 (45.1%)	106 (54.9%)	
pT stage				0.007
T1	119 (30.1%)	75 (63.0%)	44 (37.0%)	
T2	81 (20.5%)	42 (51.9%)	39 (48.1%)	
T3	181 (45.7%)	78 (43.1%)	103 (56.9%)	
T4	15 (3.8%)	6 (40.0%)	9 (60.0%)	
pN stage				0.197
N0	202 (51.0%)	103 (51.0%)	99 (49.0%)	
N1	165 (41.7%)	78 (47.3%)	87 (52.7%)	
N2	20 (5.1%)	14 (70.0%)	6 (30.0%)	
N3	9 (2.3%)	6 (66.7%)	3 (33.3%)	
pTNM stage				0.096
I	84 (21.2%)	52 (61.9%)	32 (38.1%)	
II	181 (45.7%)	88 (48.6%)	93 (51.4%)	
III	119 (30.0%)	57 (47.9%)	62 (52.1%)	
IV	12 (3.0%)	4 (33.3%)	8 (66.7%)	

TIM-3, mucin-dominant containing-3; ESCC, oesophageal squamous cell carcinoma.

[a]Never smokers, a lifetime smoking dose of fewer than 100 cigarettes; former smokers, those who have stopped smoking for more than 1 year; current smokers, those who currently smoke or have quit for less than 1 year.

[b]The high density of TIM-3 expression is defined in the main manuscript.

Supplementary Table 8. Association of PD-L1 Expression (5%) in Tumour Cell with Clinicopathological Factors in ESCC

		PD-L1 Tumour Cells 5%[b]		*P* value
Characteristics	All cases	Positive	Negative	
Total	396	89 (22.5%)	307 (77.5%)	
Age, years				0.488
<60	126 (31.8%)	31 (24.6%)	95 (75.4%)	
≥60	270 (68.2%)	58 (21.5%)	212 (78.5%)	
Sex				0.939
Male	370 (93.4%)	83 (22.4%)	287 (77.6%)	
Female	26 (6.6%)	6 (23.1%))	20 (76.9%)	
Location				0.417
Upper	24 (6.1%)	7 (29.2%)	17 (70.8%)	
Middle	84 (21.2%)	15 (18.9%)	69 (82.1%)	
Lower	288 (72.7%)	67 (23.3%)	221 (76.7%)	
Tumour grade				0.134
Well	72 (18.2%)	10 (13.9%)	62 (86.1%)	
Moderate	261 (65.9%)	62 (23.8%)	199 (76.2%)	
Poorly	63 (15.9%)	17 (27.0%)	46 (73.0%)	
Smoking status[a]				0.103
Never smoker	103 (26.0%)	19 (18.4%)	84 (81.6%)	
Former smoker	100 (100%)	30 (30.0%)	70 (70.0%)	
Current smoker	193 (48.7%)	40 (20.7%)	153 (79.3%)	
pT stage				0.821
T1	119 (30.1%)	26 (21.8%)	93 (78.2%)	
T2	81 (20.5%)	18 (22.2%)	63 (77.8%)	
T3	181 (45.7%)	43 (23.8%)	138 (76.2%)	
T4	15 (3.8%)	2 (13.3%)	13 (86.7%)	
pN stage				0.002
N0	202 (51.0%)	48 (23.8%)	154 (76.2%)	
N1	165 (41.7%)	27 (16.4%)	138 (83.6%)	
N2	20 (5.1%)	10 (50.0%)	10 (50.0%)	
N3	9 (2.3%)	4 (44.4%)	5 (55.6%)	
pTNM stage				0.373
I	84 (21.2%)	24 (28.6%)	60 (71.4%)	
II	181 (45.7%)	35 (19.3%)	146 (80.7%)	
III	119 (30.0%)	28 (23.5%)	91 (76.5%)	
IV	12 (3.0%)	2 (16.7%)	10 (83.30%)	

ESCC, oesophageal squamous cell carcinoma; PD-L1, programmed cell death-1 ligand.
[a]Never smokers, a lifetime smoking dose of fewer than 100 cigarettes; former smokers, those who have stopped smoking for more than 1 year; current smokers, those who currently smoke or have quit for less than 1 year.

[b]The positivity of PD-L1 expression in tumour cells is defined in the main manuscript.

Supplementary Table 9. Association of PD-L1 Expression (5%) in Immune Cell with Clinicopathological Factors in ESCC

		PD-L1 Immune Cells (5%)[b]		*P* value
Characteristics	All cases	Positive	Negative	
Total	396	141 (35.6%)	255 (64.4%)	
Age, years				0.186
<60	126 (31.8%)	39 (31.0%)	87 (69.0%)	
≥60	270 (68.2%)	102 (37.8%)	168 (62.2%)	
Sex				0.339
Male	370 (93.4%)	134 (36.2%)	236 (63.8%)	
Female	26 (6.6%)	7 (26.9%)	19 (73.1%)	
Location				0.356
Upper	24 (6.1%)	6 (25.0%)	18 (75.0%)	
Middle	84 (21.2%)	27 (32.1%)	57 (67.9%)	
Lower	288 (72.7%)	108 (37.5%)	180 (62.5%)	
Tumour grade				0.744
Well	72 (18.2%)	23 (31.9%)	49 (68.1%)	
Moderate	261 (65.9%)	96 (36.8%)	165 (63.2%)	
Poorly	63 (15.9%)	22 (34.9%)	41 (65.1%)	
Smoking status[a]				0.408
Never smoker	103 (26.0%)	36 (35.0%)	67 (65.0%)	
Former smoker	100 (25.2%)	41 (41.0%)	59 (59.0%)	
Current smoker	193 (48.7%)	64 (33.2%)	129 (66.8%)	
pT stage				0.253
T1	119 (30.1%)	45 (37.8%)	74 (62.2%)	
T2	81 (20.5%)	35 (43.2%)	46 (56.8%)	
T3	181 (45.7%)	57 (31.5%)	124 (68.5%)	
T4	15 (3.8%)	4 (26.7%)	11 (73.3%)	
pN stage				0.709
N0	202 (51.0%)	72 (35.6%)	130 (64.4%)	
N1	165 (41.7%)	58 (35.2%)	107 (64.8%)	
N2	20 (5.1%)	9 (45.0%)	11 (55.0%)	
N3	9 (2.3%)	2 (22.2%)	7 (77.8%)	
pTNM stage				0.284
I	84 (21.2%)	37 (44.0%)	47 (56.0%)	
II	181 (45.7%)	63 (34.8%)	118 (65.2%)	
III	119 (30.0%)	38 (31.9%)	81 (68.1%)	
IV	12 (3.0%)	3 (25.0%)	9 (75.0%)	

ESCC, oesophageal squamous cell carcinoma; PD-L1, programmed cell death-1 ligand.

[a]Never smokers, a lifetime smoking dose of fewer than 100 cigarettes; former smokers, those who have stopped smoking for more than 1 year; current smokers, those who currently smoke or have quit for less than 1 year.

[b]The positivity of PD-L1 expression in immune cells is defined in the main manuscript.

Supplementary Table 10. Correlation between Immune Markers in Immune Cells

	CD3	CD8	Foxp3	ICOS	LAG-3	PD-1	TIM-3	PD-L1
CD3	N/A	r = 0.835<br>P < 0.001	r = 0.380<br>P < 0.001	r = 0.128<br>P = 0.011	r = 0.639<br>P < 0.001	r = 0.370<br>P < 0.001	r = 0.460<br>P < 0.001	r = 0.477<br>P < 0.001
CD8		N/A	r = 0.249<br>P < 0.001	r = 0.087<br>P = 0.086	r = 0.687<br>P < 0.001	r = 0.351<br>P < 0.001	r = 0.497<br>P < 0.001	r = 0.516<br>P < 0.001
Foxp3			N/A	r = 0.241<br>P < 0.001	r = 0.261<br>P < 0.001	r = 0.322<br>P < 0.001	r = 0.277<br>P < 0.001	r = 0.205<br>P < 0.001
ICOS				N/A	r = 0.081<br>P = 0.108	r = 0.237<br>P < 0.001	r = 0.248<br>P < 0.001	r = 0.377<br>P < 0.001
LAG-3					N/A	r = 0.405<br>P < 0.001	r = 0.437<br>P < 0.001	r = 0.474<br>P < 0.001
PD-1						N/A	r = 0.328<br>P < 0.001	r = 0.307<br>P < 0.001
TIM-3							N/A	r = 0.555<br>P < 0.001

Foxp3, forkhead box P3; ICOS, inducible co-stimulator; LAG-3, lymphocyte activation gene-3; PD-1, programmed cell death protein-1; TIM-3, T-cell immunoglobulin and mucin-dominant containing-3; PD-L1, programmed death-ligand 1; *r*, correlation coefficient; N/A, not applicable.

Supplementary Figure 1. Representative immunohistochemistry stainings.

(A) A representative sample showing CD3, CD8, and regulatory T-cells: (a) low infiltration of CD3+ T-cells (b) high infiltration of CD3+ T-cells (c) low infiltration of CD8+ T-cells (d) high infiltration of CD8+ T-cells (e) low infiltration of Foxp3+ T-cells (f) high infiltration of Foxp3+ T-cells.

(B) A representative sample showing PD1, LAG-3, ICOS, and TIM-3-expressing T-cells: (a) low infiltration of PD1+ T-cells (b) high infiltration of PD1+ T-cells (c) low infiltration of LAG-3+ T-cells (d) high infiltration of LAG-3+ T-cells (e) low infiltration of ICOS+ T-cells (f) high infiltration of ICOS+ T-cells (g) low infiltration of TIM-3+ T-cells (h) high infiltration of TIM3+ T-cells.

(C) A representative sample showing PD-L1 expression on tumour and immune cells. PD-L1 expression was semi-quantitatively graded: PD-L1 expression on TC was scored as TC0, 0% (a); TC1, >0% but <5% (b); TC2, ≥5% but <10% (c); TC3, ≥10% but <25% (d); TC4, ≥25% but <50% (e); TC5, ≥50% (f). PD-L1 expression on immune cells (IC) was scored as IC0, 0% (g); IC1, >0% but <5% (h); IC2, ≥5% but <10% (i); IC3, ≥10% but <25% (j); IC4, ≥25% but <50% (k); IC5, ≥50% (l).
Foxp3, forkhead box P3; IC, immune cell; ICOS, inducible co-stimulator; LAG-3, lymphocyte activation gene-3; PD-L1, programmed cell death-ligand 1; PD-1, programmed cell death-1; TC, tumour cell; TIM-3, T-cell immunoglobulin and mucin-dominant containing-3.

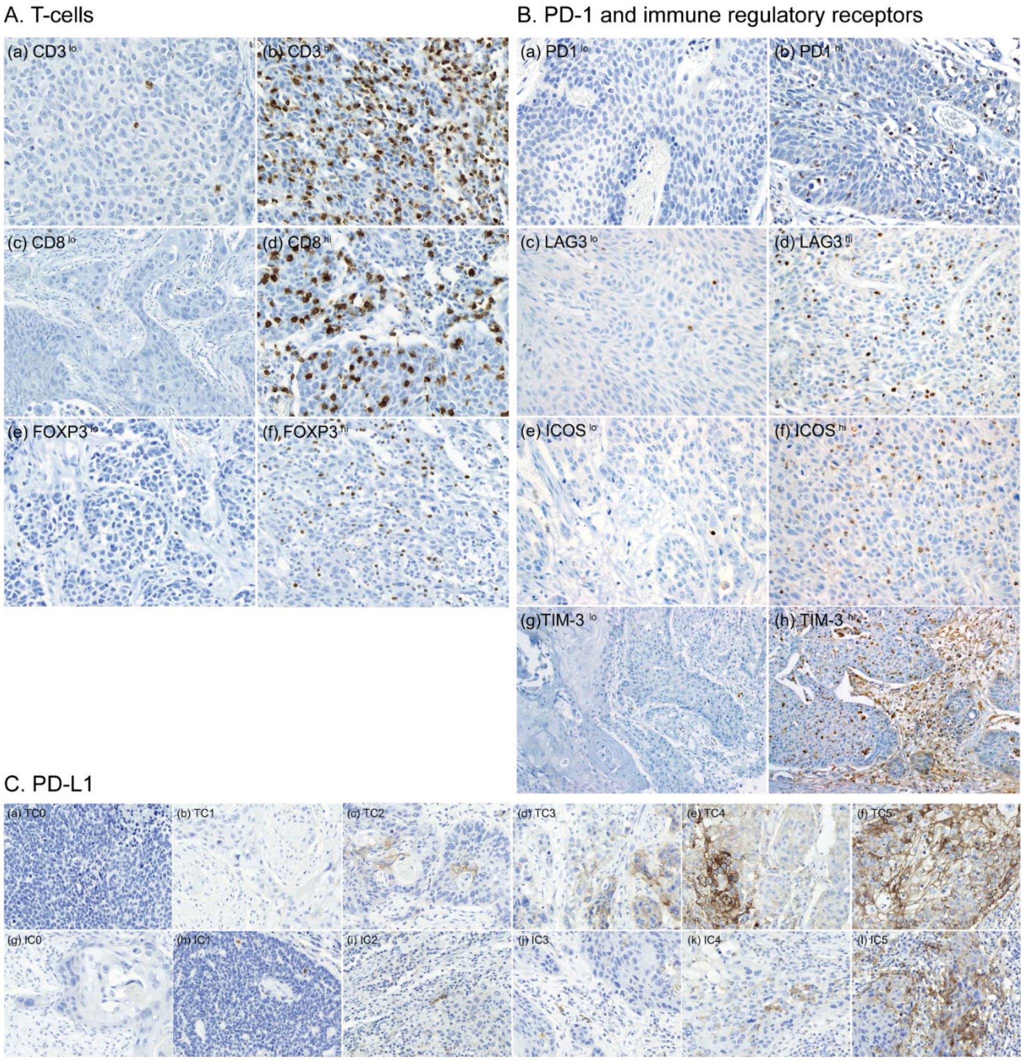

Supplementary Figure 2. Kaplan-Meier analysis for overall survival based on TGCA data set with low and high *HAVCR2* gene mRNA expression profile (n = 85).

TCGA, The Cancer Genome Atlas; *HAVCR2*, Hepatitis A virus cellular receptor 2.

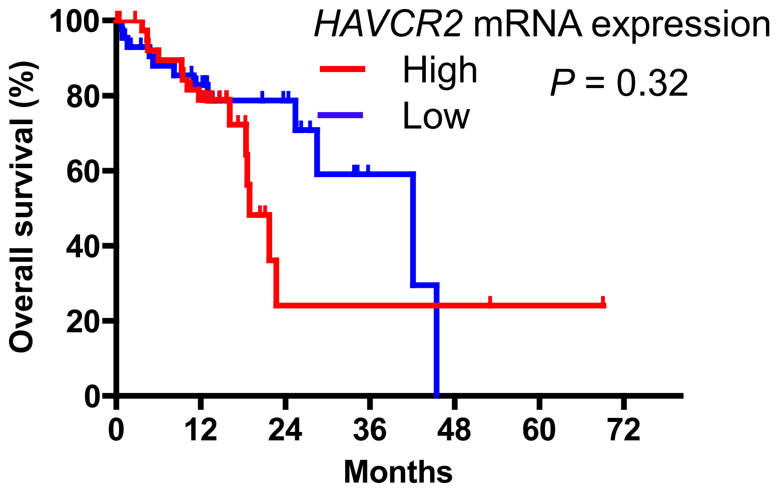

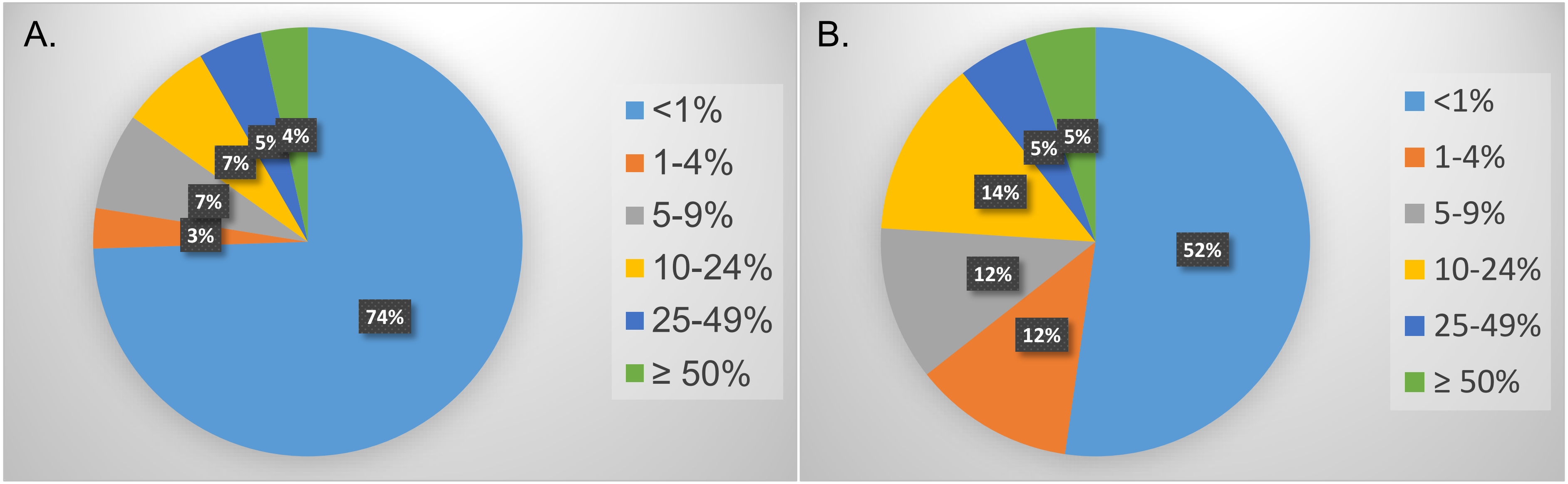

Supplementary Figure 3. Distribution of PD-L1 expression in tumour cells (A) and immune cells (B) in resected ESCC (n = 396).

ESCC, oesophageal squamous cell carcinoma.

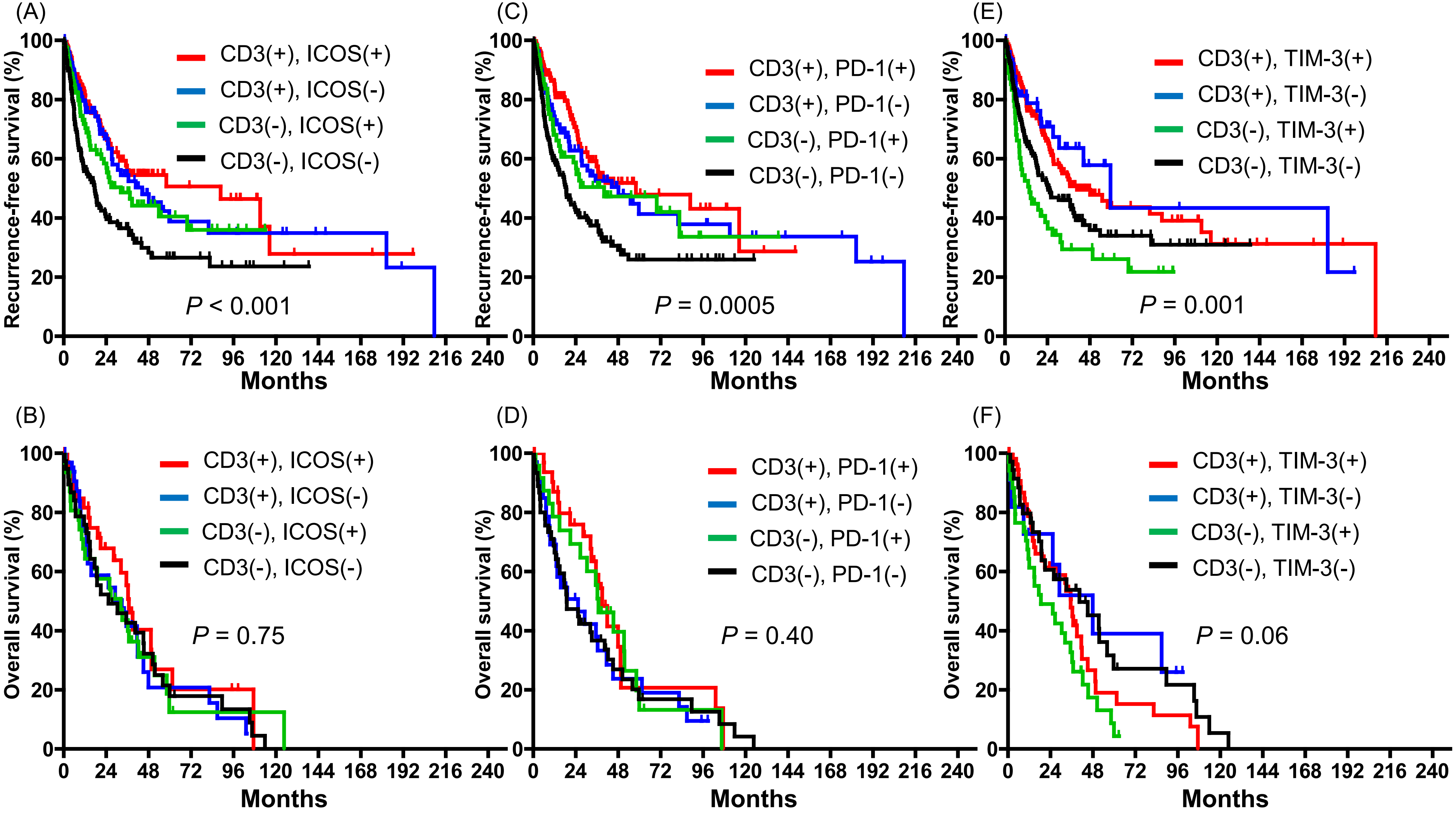

Supplementary Figure 4. Recurrence-free survival and overall survival in each subgroup.

(A) Recurrence-free survival and (B) overall survival in relation to the CD3 and ICOS expression status.
(C) Recurrence-free survival and (D) overall survival in relation to the CD3 and PD-1 expression status.
(E) Recurrence-free survival and (F) overall survival in relation to the CD3 and TIM-3 expression status.
ICOS, inducible co-stimulator; TIM-3, T-cell immunoglobulin and mucin-dominant containing-3.

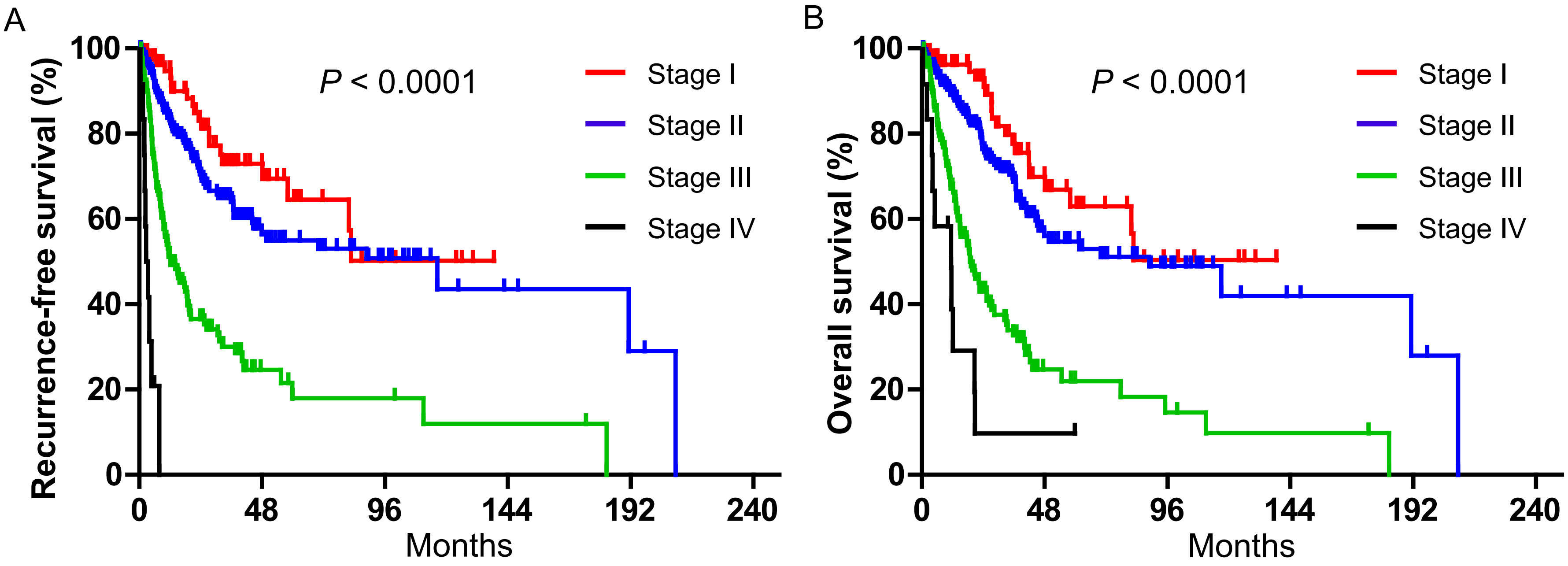

Supplementary Figure 5. Recurrence-free survival (A) and overall survival (B) according to the stage.
